# Supplementary material for: Anderson transition in stoichiometric Fe2VAl: high thermoelectric performance from impurity bands
Source: Nat Commun. 2022 Jun 23;13:3599. doi: 10.1038/s41467-022-31159-w (PMC9226177; doi:10.1038/s41467-022-31159-w)
Supplement: Supplementary file 1 — Supplementary Information [file 41467_2022_31159_MOESM1_ESM.pdf]

Supplementary Information

Anderson transition in stoichiometric Fe_2VAl: High thermoelectric performance from impurity bands

Fabian Garmroudi[1], Michael Parzer[1], Alexander Riss[1], Andrei V. Ruban[2,3], Sergii Khmelevskyi[4], Michele Reticcioli[5], Matthias Knopf[1], Herwig Michor[1], Andrej Pustogow[1], Takao Mori[6,7], Ernst Bauer[1]

[1] *Institute of Solid State Physics, Technische Universität Wien, Vienna, Austria*
[2] *Department of Materials Science and Engineering, KTH Royal Institute of Technology, Stockholm, Sweden*
[3] *Materials Center Leoben Forschung GmbH, Leoben, Austria*
[4] *Research Center for the Computational Materials Science and Engineering, Technische Universität Wien, Vienna, Austria*
[5] *Faculty of Physics, Center for Computational Materials Science, Universität Wien, Vienna, Austria*
[6] *International Center for Materials Nanoarchitectonics (WPI-MANA), National Institute for Materials Science, Tsukuba, Japan*
[7] *Graduate School of Pure and Applied Sciences, University of Tsukuba, Tsukuba, Japan*

Supplementary Note 1: Calculation of electronic charges

Hinterleitner *et al.*[1] recently calculated the charge transfer in the Fe_2VAl compound by means of Bader's quantum theory of atoms in molecules. Such Bader charge analysis of the charge transfer $\Delta q_{\mathrm{at}} = q_{\mathrm{at}}^{\mathrm{scf}} - q_{\mathrm{at}}^{\mathrm{sup}}$, with $q_{\mathrm{at}}^{\mathrm{scf}}$ and $q_{\mathrm{at}}^{\mathrm{sup}}$ being the self-consistently derived charge density and superposed atomic Bader charges respectively, showed that there occurs a significant charge transfer towards each Fe atom $\Delta q_{\mathrm{Fe}} = -0.75\,e$ from both V ($\Delta q_{\mathrm{V}} = 0.48\,e$) and Al ($\Delta q_{\mathrm{Al}} = 1.03\,e$) in Fe_2VAl. To illustrate the localised nature of Fe antisite defects, we calculated the charge density and Bader charges for the above mentioned $3 \times 3 \times 3$ rhombohedral supercell (54 Fe, 27 V, 27 Al atoms) containing one Fe/V antisite exchange defect. We found that the calculated charge transfer of Fe antisite exchanges is almost neglibile (Fe/V defect: $-0.14\,e$ for Fe and V, Fe/Al defect: $-0.02\,e$ for Fe and $0.07\,e$ for Al). This clearly shows that the charge is more localised around these defects, which act as impurities that are isolated from the ordered Fe_2VAl host compound. Our calculations are also consistent with similar calculations of single V_{Fe} antisites by Bilc *et al.*[2].

Supplementary Note 2: Reproducibility & stability of measurements

We checked the reproducibility and consistency of our results by preparing an isoelectronic Fe_2VAl-based sample by slightly changing the composition to $Fe_2V_{0.95}Ta_{0.05}Al$. The results for the measured thermopower and power factor are shown in Supplementary Fig.5. One can see that an almost identical behaviour of the thermopower upon high-temperature quenching is found further validating the consistency and reproducibility of the temperature-induced disorder in this series of full-Heusler compounds.

Mechanical strength and stability are prerequisites for building reliable thermoelectric devices. Therefore, we tested the stability of the rapidly quenched sample by measuring the thermoelectric properties during several heating and cooling cycles. We find that the temperature-dependent thermopower and power factor (see Supplementary Fig.5a,b) are surprisingly stable and did not degrade during several measurement runs, in different directions of the sample, with several heating and cooling cycles, despite the rapid quenching procedure. This confirms that the temperature-induced disorder could be a valid strategy to optimise the performance of thermoelectric devices, at least when the operating temperature is sufficiently below the quenching temperature.

Supplementary Note 3: Charge transport model for impurity band at the Anderson transition

The temperature-dependent electrical conductivity, thermopower and electronic thermal conductivity can be generally expressed as[3]

$$\sigma(T) = L_{11}, \;\; S(T) = \frac{1}{T}\frac{L_{12}}{L_{11}}, \;\; \kappa_{\rm e} = \frac{1}{T}\frac{L_{11}L_{22} - L_{12}^2}{L_{11}}, \tag{1}$$

with L_{ij} being the electronic linear response coefficients, which are given by the Chester-Thellung-Kubo-Greenwood (CTKG) formulation[4] as

$$L_{11} = \int_{-\infty}^{\infty} \Sigma(E,T)\left(-\frac{\partial f(E,T)}{\partial E}\right) dE, \tag{2}$$

$$L_{12} = \frac{1}{|e|}\int_{-\infty}^{\infty} \Sigma(E,T)(E-\mu)\left(-\frac{\partial f(E,T)}{\partial E}\right) dE, \tag{3}$$

$$L_{22} = \frac{1}{e^2}\int_{-\infty}^{\infty} \Sigma(E,T)(E-\mu)^2\left(-\frac{\partial f(E,T)}{\partial E}\right) dE. \tag{4}$$

Hereby, $\Sigma(E,T)$ denotes an energy-dependent transport function that depends on the physics of the system. It was shown that $\Sigma(E,T)$ near the mobility edge or more generally near the transport edge[5] of partly localised electronic systems, usually follows

$$\Sigma(E,T) = \Sigma_0(T)\left(\frac{E-E_{\rm c}}{k_{\rm B}T}\right)^s, \tag{5}$$

where $\Sigma_0(T)$ is an energy-independent prefactor, $E_{\rm c}$ is the critical energy for delocalisation, *i.e.*, the mobility or transport edge and s is an exponent that determines the shape of the transport function and is usually material-dependent. For the Anderson transition the critical exponent s is between 0.5 and 2 depending on compensation and band hybridisation[6]. We extended this concept to impurity bands with two mobility edges (see Supplementary Fig.9a) and developed a model where the transport function increases following Eq.5 at the first mobility edge and decreases in the same manner at the second mobility edge. Thus, the total energy dependence of the transport function is given by its width W, height Σ_0 and exponent s (see Supplementary Fig.9a). By numerically evaluating the integrals in Eqs.2−4, we can model the temperature dependencies of the thermoelectric transport properties from such an impurity band. For normal parabolic bands with dominant acoustic phonon scattering the transport properties can be written as[7,8]

$$\sigma(T) = \sigma_0(T)F_0(\eta,T), \tag{6}$$

$$S(T) = \frac{k_{\rm B}}{e}\left[\eta - \frac{F_1(\eta,T)}{F_0(\eta,T)}\right], \tag{7}$$

$$\kappa_{\rm e}(T) = \frac{3F_0(\eta,T)F_2(\eta,T) - 4F_1^2(\eta,T)}{F_0^2(\eta,T)}\sigma(T)T, \tag{8}$$

where $\eta = \frac{E-\mu}{k_{\rm B}T}$ is the reduced chemical potential and $F_j(\eta,T)$ represent the Fermi integrals[9]

$$F_j(\eta,T) = \int_0^{\infty} \frac{\xi^j}{1+\exp(\xi-\eta)} d\xi. \tag{9}$$

In contrary to σ and κ the thermopower does not depend on the absolute magnitude of the transport function, *i.e.*, the transport height. The same is true for the parabolic band expression where one can see that $S(T)$ only depends on the Fermi integrals. The electrical conductivity on the other hand includes an energy-independent $\sigma_0(T)$, which does not cancel out and holds information about the band masses,

band degeneracies and scattering mechanisms. Thus, when modelling temperature-dependent transport, one should start by modelling the thermopower, which is a more direct probe of the energy-dependent electronic structure and significantly reduces the number of model parameters [10]. We started by making a qualitative analysis and simulated the temperature-dependent thermopower with a simple parabolic two band model with a tiny band gap, where the Fermi level is positioned near the valence band edge. Such scenario is expected to be likely the case for the ordered Fe_2VAl compound [1,11]. We then introduced an impurity band near E_F and increased the width of its extended states, *i.e.*, delocalised it. We already found a good qualitative agreement by just increasing the transport width (delocalisation), which gives the sign reversal of the thermopower over the whole temperature range without even shifting the Fermi level or adjusting the band masses. To further test the applicability of our model towards the measured data, we developed a least-squares fit model. Supplementary Fig.9b shows the measured temperature-dependent thermopower from 4 K to 800 K together with least-squares fits from a simple two-parabolic band model as well as our extended model containing the impurity band. A remarkable agreement and improvement over the entire temperature range is found for the latter, while the former yields poor agreement, especially at higher temperatures. The inset shows the predicted thermopower at room temperature as a function of the reduced chemical potential, predicting a sign reversal due to the delocalised impurity band, which was confirmed in our experiments. By continuously varying the disorder parameter W, which corresponds to the width of the electronic transport function, we are able to reproduce our experimentally observed trends of the thermopower almost perfectly, even though the energy gaps are only slightly varied. Our model confirms that W approaches zero for a quenching temperature $T_{\text{quench}} \approx 950 - 1050\,^{\circ}\text{C}$, which is consistent with all conclusions drawn from our resistivity and Hall effect experiments.

Supplementary References

[1] Hinterleitner, B. *et al.* The electronic pseudo band gap states and electronic transport of the full-Heusler compound Fe_2VAl. *Journal of Materials Chemistry C* **9**, 2073–2085 (2021).

[2] Bilc, D. I. & Ghosez, P. Electronic and thermoelectric properties of Fe_2VAl: the role of defects and disorder. *Physical Review B* **83**, 205204 (2011).

[3] Yamamoto, K., Aharony, A., Entin-Wohlman, O. & Hatano, N. Thermoelectricity near Anderson localization transitions. *Physical Review B* **96**, 155201 (2017).

[4] Chester, G. & Thellung, A. The law of Wiedemann and Franz. *Proceedings of the Physical Society (1958-1967)* **77**, 1005 (1961).

[5] Kang, S. D. & Snyder, G. J. Charge-transport model for conducting polymers. *Nature Materials* **16**, 252–257 (2017).

[6] Carnio, E. G., Hine, N. D. & Römer, R. A. Resolution of the exponent puzzle for the Anderson transition in doped semiconductors. *Physical Review B* **99**, 081201 (2019).

[7] Kim, H.-S., Gibbs, Z. M., Tang, Y., Wang, H. & Snyder, G. J. Characterization of Lorenz number with Seebeck coefficient measurement. *APL Materials* **3**, 041506 (2015).

[8] May, A. F. & Snyder, G. J. Introduction to modeling thermoelectric transport at high temperatures. In *Materials, Preparation, and Characterization in Thermoelectrics*, 11–1 (CRC press, 2017).

[9] Kim, R., Wang, X. & Lundstrom, M. Notes on Fermi-Dirac integrals. *arXiv preprint arXiv:0811.0116* (2008).

[10] Garmroudi, F. *et al.* Boosting the thermoelectric performance of Fe_2VAl-type Heusler compounds by band engineering. *Physical Review B* **103**, 085202 (2021).

[11] Anand, S. *et al.* Thermoelectric transport of semiconductor full-Heusler VFe_2Al. *Journal of Materials Chemistry C* **8**, 10174–10184 (2020).

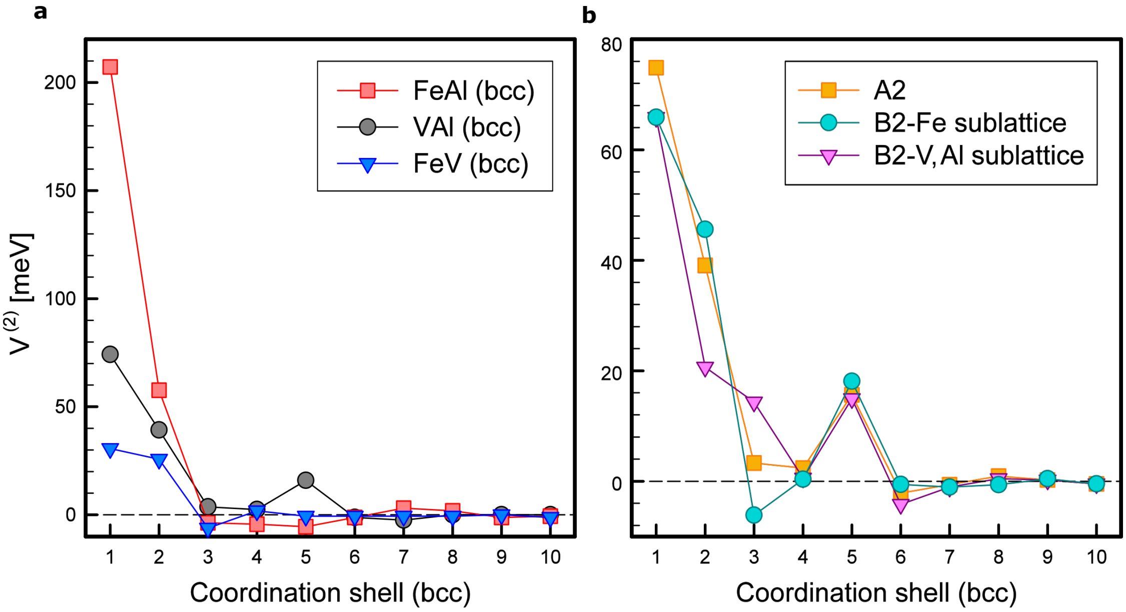

Supplementary Fig. 1 **a** Calculated effective pair interactions in the bcc $Fe_{0.5}V_{0.25}Al_{0.25}$ alloy. **b** V-Al effective pair interactions in the bcc $Fe_{0.5}V_{0.25}Al_{0.25}$ alloy and in the B_2 partially ordered alloy on the Fe and V$-$Al sublattices $\cdot$

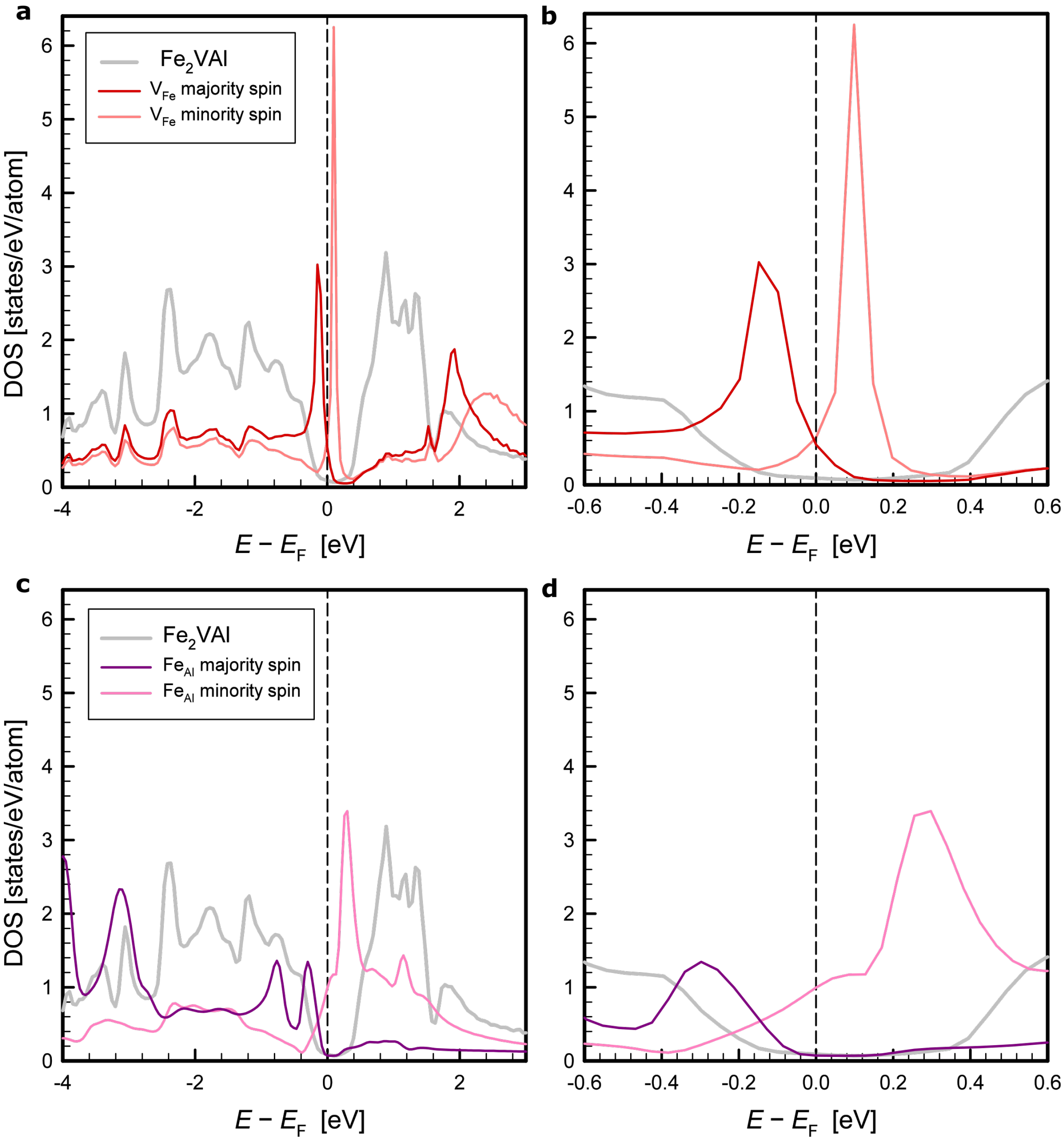

Supplementary Fig. 2 **a**,**b** Spin-polarised density of states (DOS) of the single-impurity V_{Fe} and **c**,**d** Fe_{Al} antisite defects, compared to the DOS of pristine Fe_2VAl; calculated within the EMTO-CPA method.

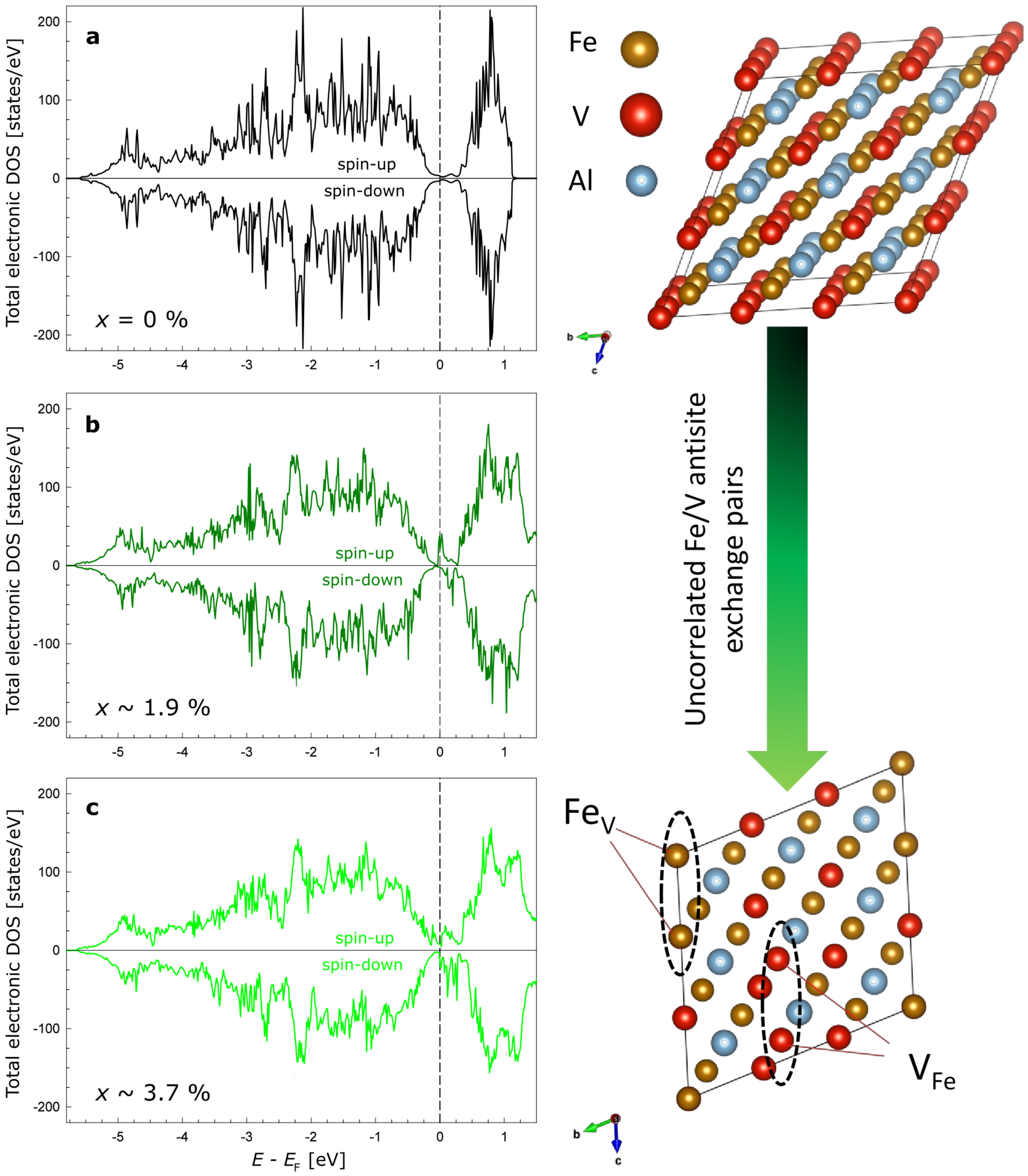

Supplementary Fig. 3 Total electronic density of states (DOS) for both spin channels of **a** the pristine $3 \times 3 \times 3$ primitive supercell (108 atoms) of Fe_2VAl **b** with one uncorrelated Fe/V antisite exchange defect (defect concentration: $x \approx 1.9\,\%$) and **c** with two uncorrelated Fe/V antisite exchange defects ($x \approx 3.7\,\%$); Supercell electronic structure calculations were calculated in VASP using standard GGA-PBE functionals. A broadening of the localised in-gap states occurs when increasing the defect concentration. Sketch (right panel) shows the supercells used for the calculations.

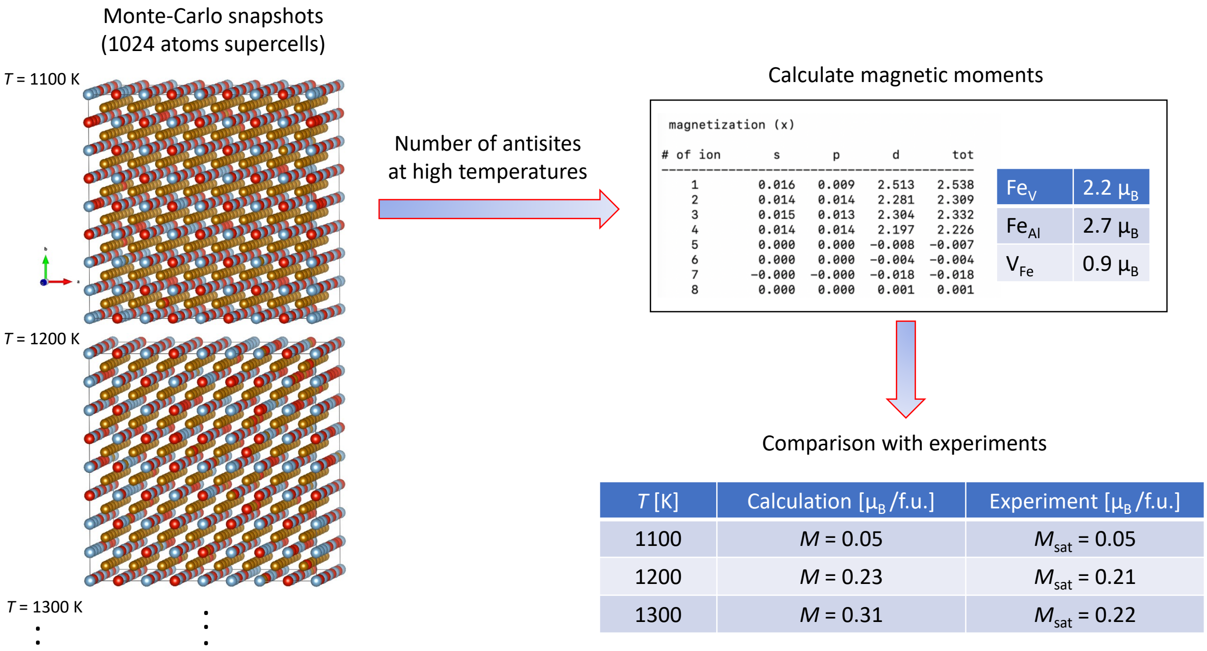

Supplementary Fig. 4 Schematic of the procedure for calculating the magnetisation of high-temperature-disordered Fe_2VAl. 1024 atoms supercells were created from Monte-Carlo snapshots of high-temperature-disordered Fe_2VAl. The number of antisites were evaluated and their magnetic moments were calculated in VASP and compared with experimental data, yielding fairly good agreement.

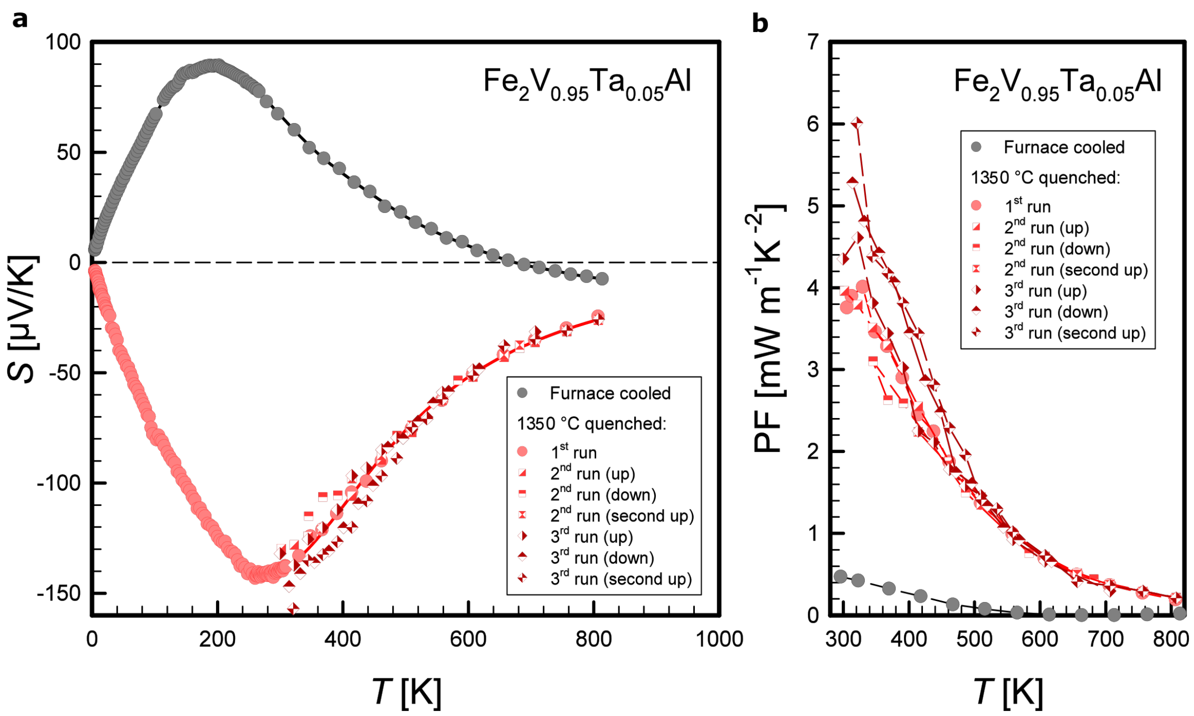

Supplementary Fig. 5 a Measured thermopower of $Fe_2V_{0.95}Ta_{0.05}Al$, furnace cooled and quenched at 1350 °C. A sign reversal, similar to the one shown in the main article for Fe_2VAl, could be observed illustrating the reproducibility of this effect. Reasonably consistent results from various measurement runs above room temperatures with several heating and cooling cycles confirm a surprisingly good stability for the thermopower of the quenched sample. **b** Power factor of $Fe_2V_{0.95}Ta_{0.05}Al$ from various measurement runs above room temperatures with several heating and cooling cycles.

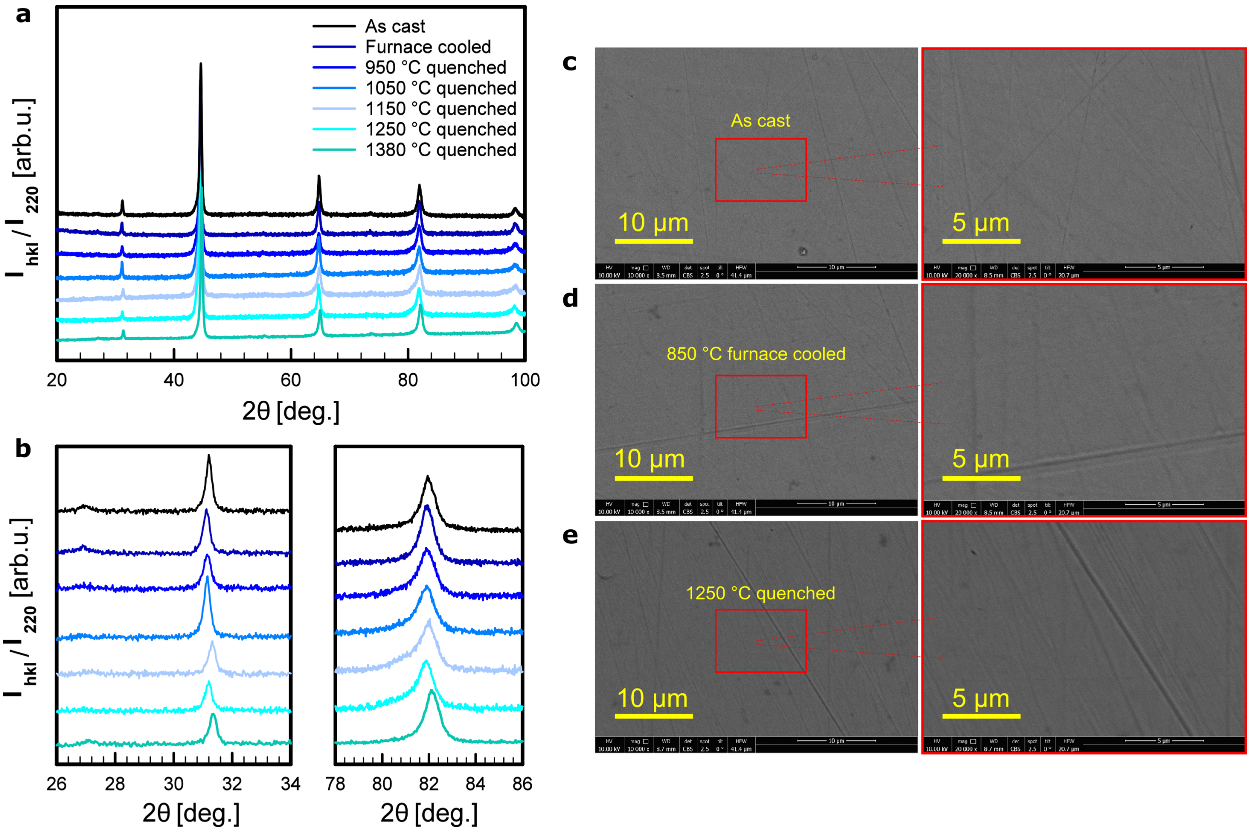

Supplementary Fig. 6 **a** Normalised intensities of XRD powder patterns for Fe_2VAl samples analysed in this work. **b** Magnificantions of the (111) peak at $\approx 27\,^\circ$ and (200) peak at $\approx 31\,^\circ$ in the left panel as well as the (422) peak at approximately $\approx 82\,^\circ$ in the right panel. **c**−**e** Scanning electron microscopy images of a polished surface for as-cast, furnace-cooled and $1250\,^\circ$C-quenched Fe_2VAl. Images were taken at different magnificantions using a back-scattered electron detector to search for potential impurity phase precipitation.

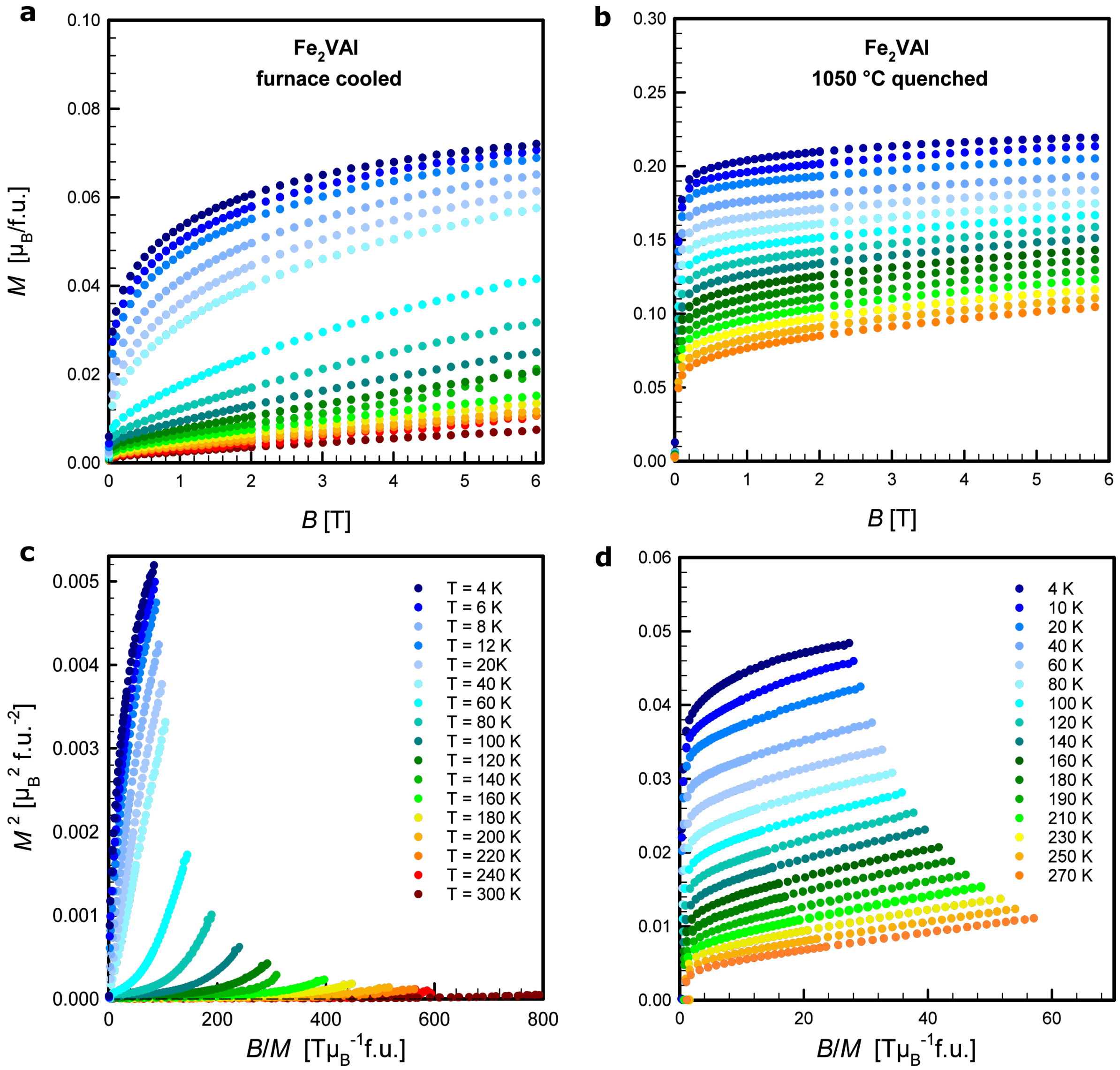

Supplementary Fig. 7 **a**,**b** Isothermal, field-dependent magnetisation curves for furnace-cooled and 1050 °C-quenched Fe_2VAl, respectively. **c**,**d** Corresponding Arrot plots for furnace-cooled and 1050 °C-quenched Fe_2VAl.

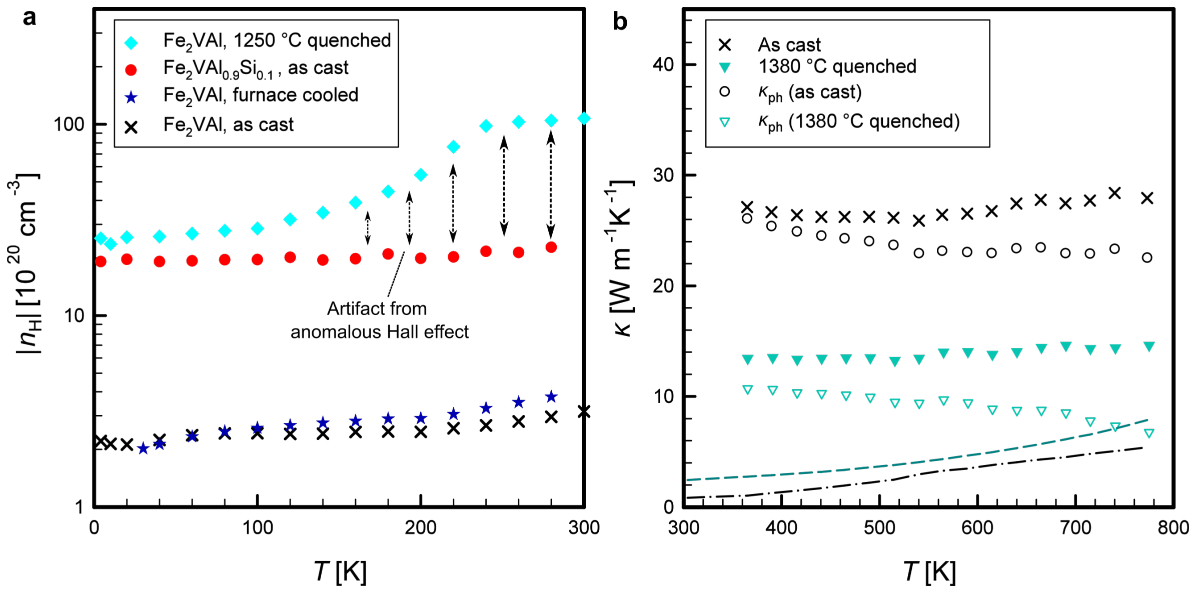

Supplementary Fig. 8 a Absolute temperature-dependent Hall carrier concentration for as-cast, furnace-cooled and 1250 °C-quenched Fe_2VAl compared to as-cast $Fe_2VAl_{0.9}Si_{0.1}$. The increase of the evaluated carrier concentration above $\approx$ 100 K for 1250 °C-quenched Fe_2VAl is presumably a measurement artifact due to the anomalous Hall effect. **b** Temperature-dependent thermal conductivity with electronic and phonon contributions of as-cast and 1380 °C-quenched Fe_2VAl. Dashed lines represent the electronic contribution κ_e.

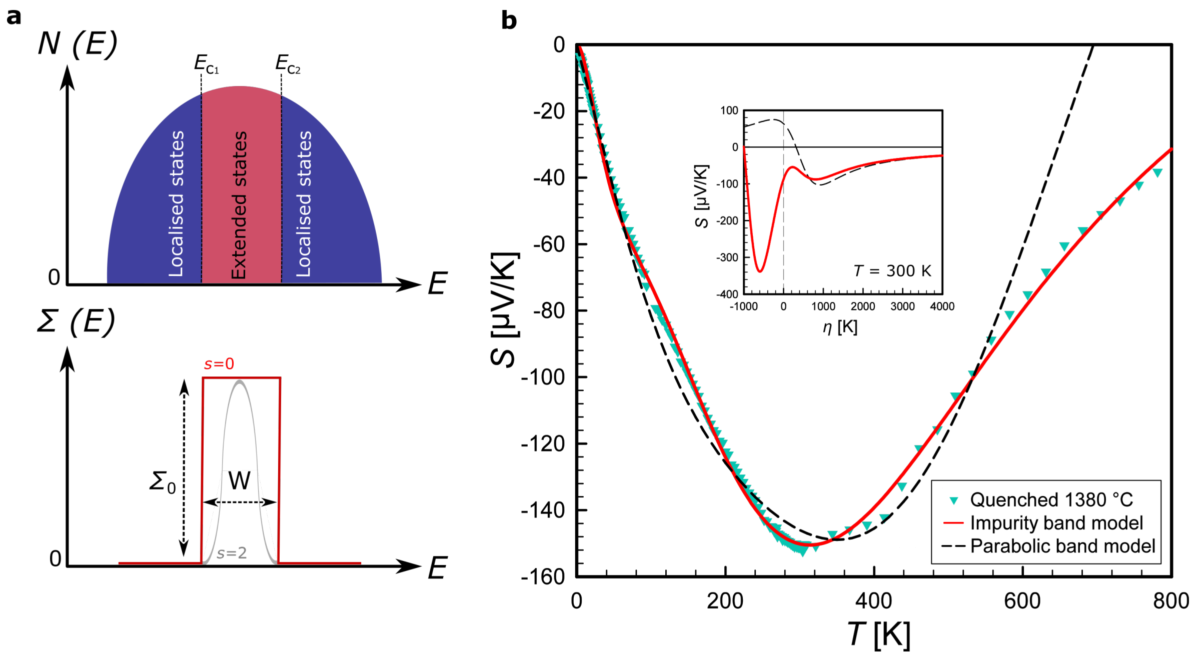

Supplementary Fig. 9 a Schematic density of states $N(E)$ for the impurity band with Anderson-localised band tails and extended states in the center of the band. The width of the extended states is given by the mobility edges E_{c_1} and E_{c_2}. The energy-dependent transport function $\Sigma(E)$ with a transport width $W = |E_{c_2} - E_{c_1}|$ takes only finite values inside the extended states (*i.e.* within the delocalised regime). **b** Temperature-dependent thermopower of 1380 °C-quenched Fe_2VAl from our experiments modelled with a parabolic two-band model and an enhanced model including the impurity band. The inset shows the predicted thermopower as a function of the reduced chemical potential, which gives a sign reversal due to the impurity contribution.

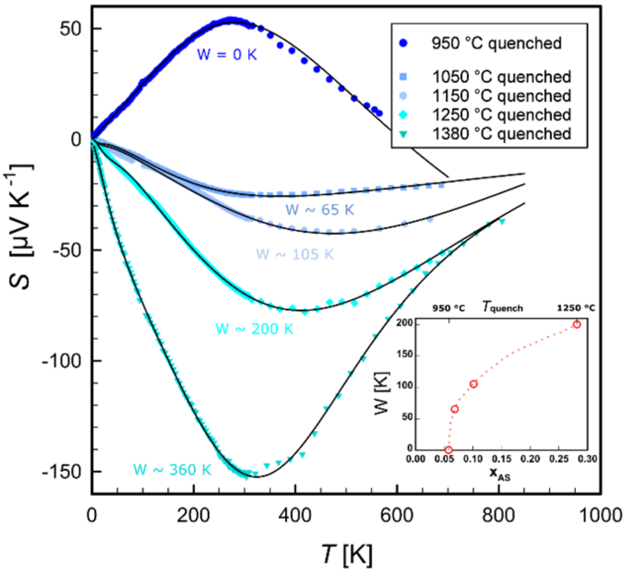

Supplementary Fig. 10 Temperature-dependent thermopower and least-squares fit model results. With increasing disorder, the width of the delocalized states of the narrow impurity band increases and reproduces the observed temperature-dependent behaviour of the thermopower.

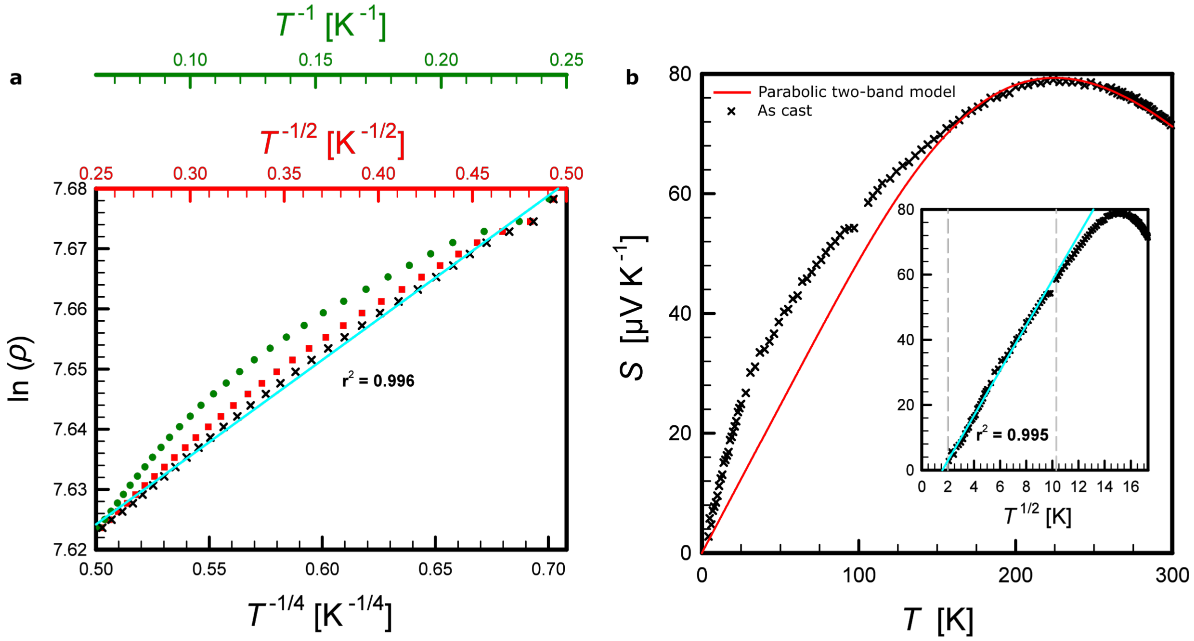

Supplementary Fig. 11 **a** Temperature-dependent behaviour of the electrical resistivity below $T = 16\,\mathrm{K}$ for as-cast Fe_2VAl. Variable range hopping conduction can best describe the low-temperature upturn in the electrical resistivity as compared to other considered activation laws. **b** Temperature-dependent thermopower of as-cast Fe_2VAl below 300 K. Inset shows an excellent fit to a square-root tempendence of $S(T)$. Red solid line indicates the temperature-dependence expected from a parabolic two-band model analysis, which linearly approaches 0 as $T \to 0\,\mathrm{K}$.

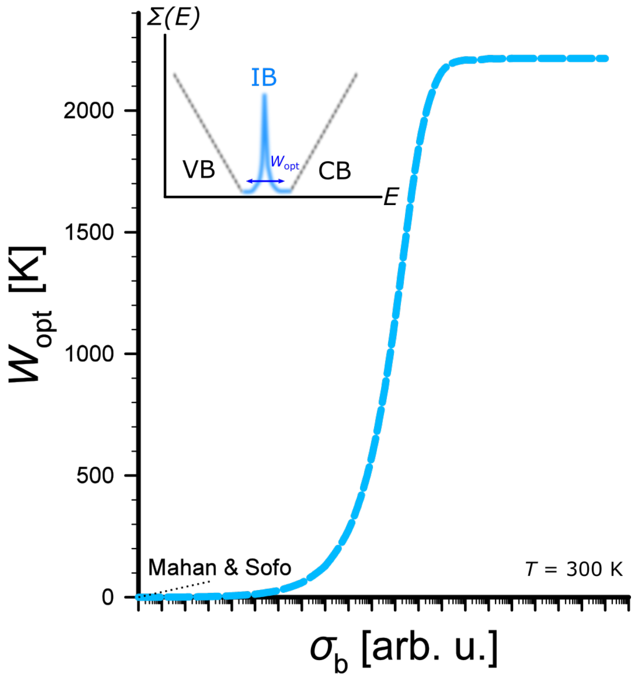

Supplementary Fig. 12 Calculated optimal energy width of the thermoelectric transport function as a function of the "background" conductivity contributions, *e.g.*, from valence and conduction bands in a semiconductor. When the electronic states of the pristine material contribute strongly to the total electrical conductivity the optimal energy width increases, whereas a delta-distribution-like transport function (Mahan and Sofo) yields the best thermoelectric performance when the conductivity of the background is neglibile. Inset shows a sketch of the transport function of valence and conduction bands as well as the transport function of a narrow delocalised impurity band at the metallic side of the Anderson transition.
